# OTUB1-mediated deubiquitination of FOXM1 up-regulates ECT-2 to promote tumor progression in renal cell carcinoma

**DOI:** 10.1186/s13578-020-00408-0

**Published:** 2020-03-30

**Authors:** Kai Zhou, Haixing Mai, Song Zheng, Weizhong Cai, Xu Yang, Zhenlin Chen, Bin Zhan

**Affiliations:** 1 Department of Urology, Fujian Medical University Union Hospital, 29 Xinquan Road, Fuzhou, 350001 Fujian China; 2 Department of Urology, Chinese PLA General Hospital, Beijing, 100853 China

**Keywords:** OTUB1, FOXM1, ECT-2, Renal cell carcinoma, Progression

## Abstract

**Background:**

OTUB1 (ovarian tumor domain protease domain-containing ubiquitin aldehyde-binding proteins)-mediated deubiquitination of FOXM1 (Forkhead box M1) participates in carcinogenesis of various tumors. We aim to investigate the effect and mechanism of OTUB1/FOXM1 on RCC (renal cell carcinoma) progression. Expression levels of OTUB1 in RCC tissues and cell lines were examined by qRT-PCR (quantitative real-time polymerase chain reaction) and immunohistochemistry. Cell proliferation was measured with CCK8 (Cell Counting Kit-8) and colony formation assays. Wound healing and transwell assays were used to determine cell migration and invasion, respectively. The effect of OTUB1 on FOXM1 ubiquitination was examined by Immunoprecipitation. Western blot was used to uncover the underlying mechanism. In vivo subcutaneous xenotransplanted tumor model combined with immunohistochemistry and western blot were used to examine the tumorigenic function of OTUB1.

**Results:**

OTUB1 was up-regulated in RCC tissues and cell lines, and was associated with poor prognosis of RCC patients. Knockdown of OTUB1 inhibited cell viability and proliferation, as well as migration and invasion of RCC cells. Mechanistically, knockdown of OTUB1 down-regulated FOXM1 expression by promoting its ubiquitination. Down-regulation of FOXM1 inhibited ECT2 (epithelial cell transforming 2)-mediated Rho signaling. Moreover, the inhibition of RCC progression caused by OTUB1 knockdown was reversed by FOXM1 over-expression. In vivo subcutaneous xenotransplanted tumor model also revealed that knockdown of OTUB1 could suppress in vivo RCC growth via down-regulation of FOXM1-mediated ECT2 expression.

**Conclusions:**

OTUB1-mediated deubiquitination of FOXM1 up-regulates ECT-2 to promote tumor progression in RCC, providing a new potential therapeutic target for RCC treatment.

## Background

Renal cell carcinoma (RCC) accounts for about 3% of all tumors with mortality rate as high as 40% [1, 2]. With steadily increasing incidence [3], there is urgent need to find novel targets for diagnosis and treatment of RCC. Although current treatments for RCC such as surgical resection or drug targeted therapies have improved tremendously, the lack of effective early diagnostic biomarkers reduces overall survival rates [4]. Moreover, due to high invasiveness and relapse rate, the mortality of RCC appears to be increasing rapidly in the past decade [2]. Therefore, identification of new sensitive diagnostic biomarkers and investigation of the underlying molecular mechanism of new therapeutic targets possess great clinical significance for improving survival rate of RCC patients.

Ubiquitination is a post-translational modification via attachment of ubiquitin on lysine residues of the targets [5]. Deubiquitinating enzymes (DUBs) are cysteine proteases that remove ubiquitin from ubiquitinated proteins [6]. DUBs have been widely known as critical regulators in tumor development and progression [7], especially in RCC [8]. Ovarian tumor (OTU)-containing DUBs is one of the members of DUBs [9] and OTUB1 (ovarian tumor domain protease domain-containing ubiquitin aldehyde-binding proteins) is a member of OTU domain protease superfamily of DUBs that removes ubiquitin from branched polyubiquitin chains in the target molecules [10]. At present, relevant studies have shown that OTUB1 plays an important regulatory role in various physiological and pathological processes such as DNA damage repair, apoptosis and inflammatory response [11–14]. Recently, the role of OTUB1 on tumorigenesis has been the focus of functional research. Studies have shown that OTUB1 is closely related to the occurrence and development of hepatocellular carcinoma [15], colorectal cancer [16], esophageal squamous cell carcinoma [17], prostate cancer [18], gastric cancer [19] and lung cancer [20]. However, the regulation ability and underlying mechanism of OTUB1 on RCC have not been reported yet.

FOXM1 (Forkhead box M1) functions as a transcriptional factor to regulate expression of proliferation-associated genes and participates in DNA replication and mitosis [21]. FOXM1 has been shown to regulate cell cycle during progression of prostate cancer [22], breast cancer [23], colorectal cancer [24] and RCC [25]. More interestingly, OTUB1 was shown to promote deubiquitination of FOXM1 in breast cancer [26] and ovarian cancer [27] to facilitate tumor progression. Therefore, we hypothesized that OTUB1-mediated deubiquitination of FOXM1 might also participate in RCC progression. We investigated the effect of OTUB1/FOXM1 axis on RCC progression and uncovered the underlying mechanism. Our study may serve as a foundation for the development of novel RCC therapy.

## Results

### OTUB1 was elevated in RCC tissues and cell lines

**Fig. 1:**
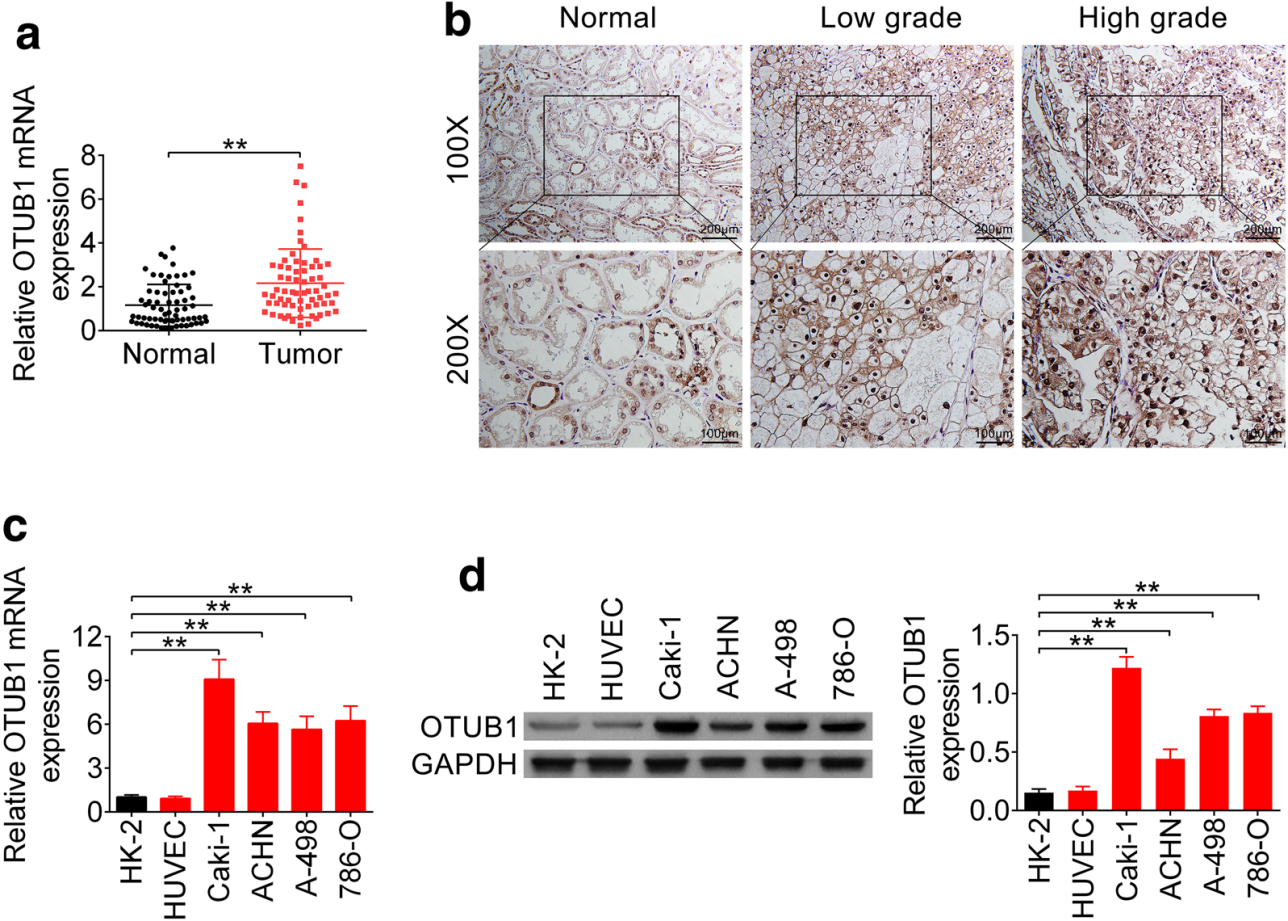
OTUB1 was elevated in RCC tissues and cell lines. **a** The expression levels of OTUB1 in RCC tissues and adjacent noncancer tissues were detected by qRT-PCR (N = 67). ** represents Tumor vs. Normal tissues, *P *< 0.01. **b** Immunohistochemistry analysis of adjacent noncancer tissues and low grade (I + II) or high grade (III + IV) RCC tissues. **c** The expression levels of OTUB1 in RCC cell lines (Caki-1, ACHN, A-498 and 786-O), HK2 and HUVEC were detected by qRT-PCR (N = 67). ** represents RCC cell lines vs. HK2, *P *< 0.01. **d** The expression levels of OTUB1 in RCC cell lines (Caki-1, ACHN, A-498 and 786-O), HK2 and HUVEC were detected by western blot. ** represents RCC cell lines vs. HK2, *P *< 0.01

To explore the correlation between OTUB1 and RCC, we analyzed the expression level of OTUB1 in RCC tissues and cell lines. Using qRT-PCR analysis, we found that OTUB1 was highly expressed in RCC tumor tissues compared to adjacent non-cancer specimens (Fig. 1a). Moreover, immunohistochemistry showed that OTUB1 expression was positively correlated with the TNM stage of RCC (Fig. 1b), suggesting that OTUB1 may contribute to RCC progression. Further analysis of correlation between OTUB1 and clinic pathologic characteristics of RCC patients indicated that among the 67 patients, high expression of OTUB1 (N = 34) was significantly correlated with T stage (*P* = 0.007), N stage (*P* = 0.026) and M stage (*P* = 0.019) (Table 1). Moreover, other clinical features such as histological grade (*P* < 0.001) and TNM stage (*P* = 0.006) were dramatically correlated with high OTUB1 expression, in consistent with immunohistochemistry analysis. However, gender (*P* = 0.365) and age (*P* = 0.393) showed no significant correlation with OTUB1 expression (Table 1). Elevation of OTUB1 was associated with poor prognosis of RCC, suggesting a potential ability of OTUB1 to serve as a prognostic biomarker for RCC. Consistent with expression in RCC tissues, OTUB1 was also elevated in RCC cell lines (Caki-1, ACHN, A-498 and 786-O) compare to HK2 and HUVEC (Fig. 1c, d). Caki-1 and 786-O cells with higher expression of OTUB1 were selected for the following functional assays.

**Table 1:** Association between OTUB1 expression and patients’ clinicopathological features

Variable	Total	OTUB1 expression		*P* value
		Low expression (< median)	High expression (≥ median)	
Number	67	33	34	
Gender				0.365
Male	41	22	19	
Female	26	11	15	
Age (years)				0.393
< 57	33	18	15	
≥ 57	34	15	19	
T stage				0.007*
T_1–2_	49	29	20	
T_3–4_	18	4	14	
N stage				0.026*
N_0_	51	29	22	
N_1–2_	16	4	12	
M stage				0.019*
M_0_	53	30	23	
M_1_	14	3	11	
Histological grade				0.000*
G_1–2_	42	28	14	
G_3–4_	25	5	20	
TNM stage				0.006*
I–II	44	27	17	
III–IV	23	6	17	

*p < 0.05

### Knockdown of OTUB1 suppressed cell proliferation, migration and invasion of RCC

**Fig. 2:**
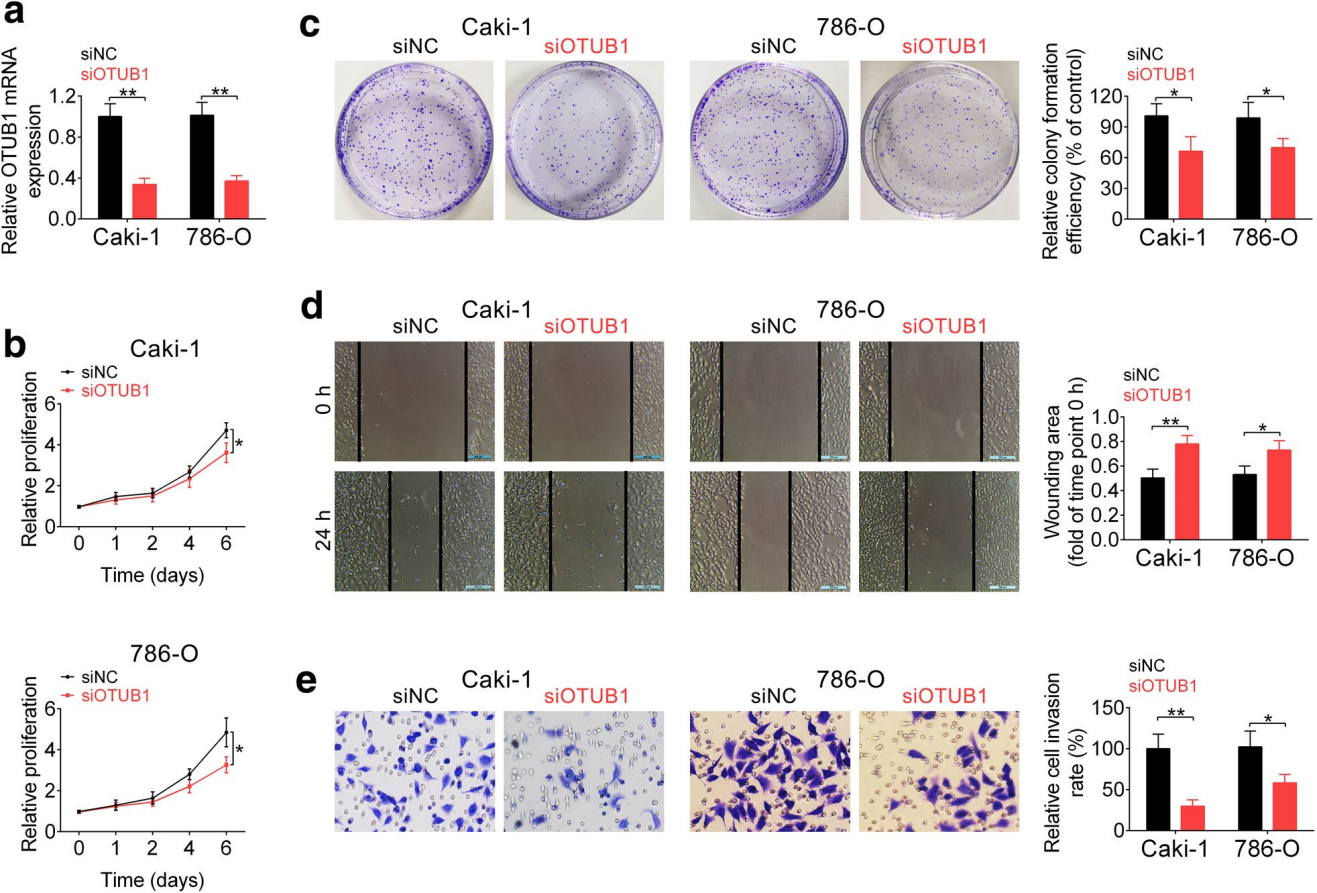
Knockdown of OTUB1 suppressed cell proliferation, migration and invasion of RCC. **a** Knockdown efficiency of siOTUB1 in Caki-1 and 786-O cells as measured by qRT-PCR. ** represents siOTUB1 vs. siNC, *p* < 0.01. **b** The effect of OTUB1 knockdown on cell viability of Caki-1 and 786-O cells. * represents siOTUB1 vs. siNC, *p* < 0.05. **c** The effect of OTUB1 knockdown on cell proliferation of Caki-1 and 786-O cells. * represents siOTUB1 vs. siNC, *p* < 0.05. **d** The effect of OTUB1 knockdown on cell migration of Caki-1 and 786-O cells. *, ** represents siOTUB1 vs. siNC, *p* < 0.05, *p* < 0.01. **e** The effect of OTUB1 knockdown on cell invasion of Caki-1 and 786-O cells. *, ** represents siOTUB1 vs. siNC, *p* < 0.05, *p* < 0.01

Loss-of function assays were conducted to determine the effects of OTUB1 on RCC progression. Two siRNAs targeting OTUB1 (siOTUB1 #1 and #2) were designed. The knockdown efficiency was confirmed in Additional file 1: Figure S1A. SiOTUB1 #1 induced stronger down-regulation of OTUB1. Therefore we selected SiOTUB1 #1 for the functional assays and renamed it as siOTUB1. The knockdown efficiency of siOTUB1 in Caki-1 and 786-O cells was confirmed using qRT-PCR in Fig. 2a. Data from CCK8 (Fig. 2b) and colony formation assay (Fig. 2c) indicated that knockdown of OTUB1 decreased cell viability and inhibited cell proliferation of RCC cells. Moreover, knockdown of OTUB1 suppressed cell migration (Fig. 2d) and invasion (Fig. 2e) of RCC, suggesting that OTUB1 might contribute to cell proliferation and malignant phenotypes of RCC. Furthermore, knockdown of OTUB1 via siOTUB1 #2 also suppressed cell viability Additional file 1: Figure S1B), proliferation (Additional file 1: Figure S1C) and migration (Additional file 1: Figure S1D) of RCC.

### OTUB1 suppressed ubiquitination of FOXM1 in RCC

**Fig. 3:**
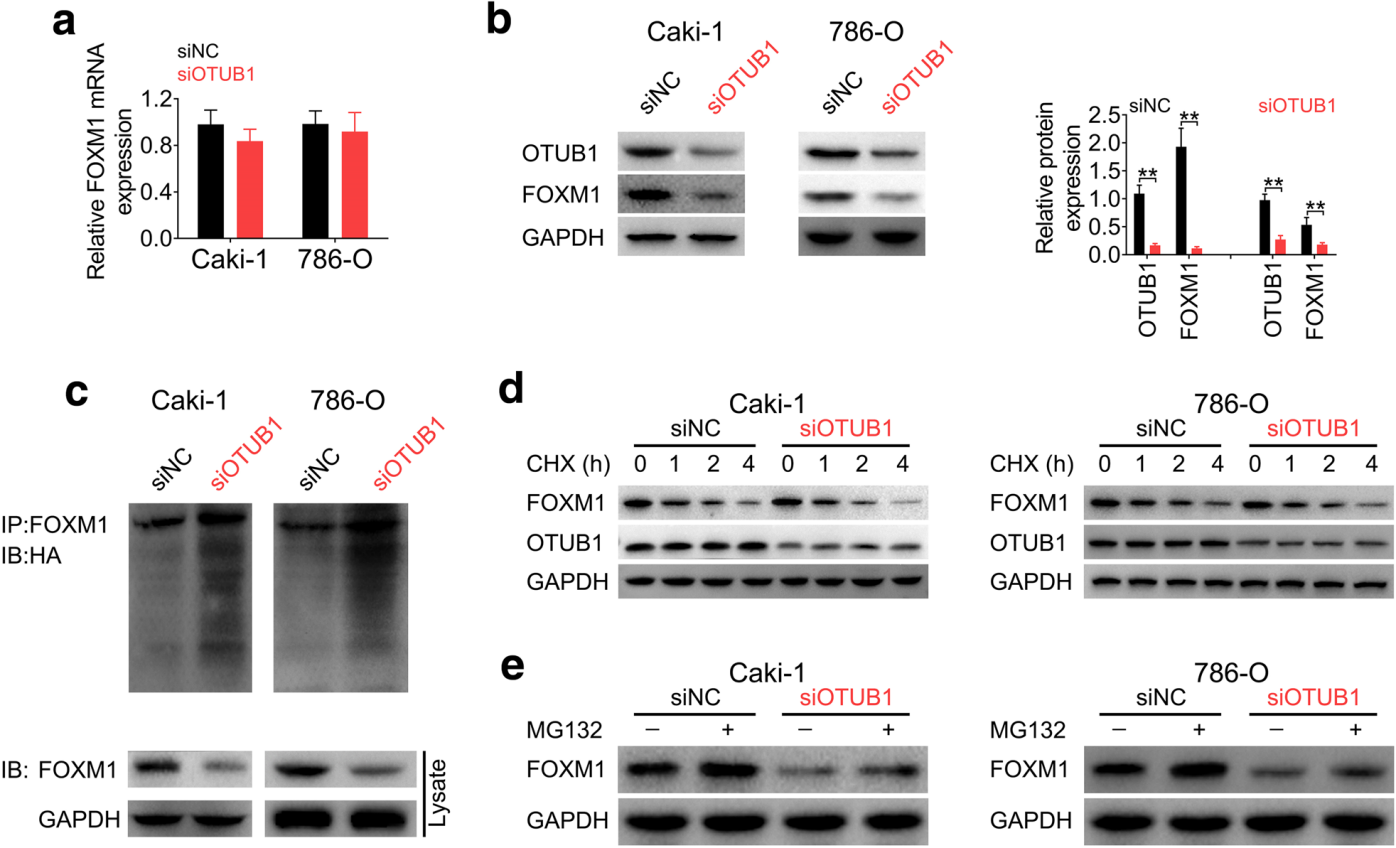
OTUB1 suppressed ubiquitination of FOXM1 in RCC. **a** The effect of OTUB1 knockdown on FOXM1 mRNA expression in Caki-1 and 786-O cells. **b** The effect of OTUB1 knockdown on FOXM1 protein expression in Caki-1 and 786-O cells. ** represents siOTUB1 vs. siNC, *p* < 0.01. **c** The effect of OTUB1 knockdown on FOXM1 ubiquitination in Caki-1 and 786-O cells. **d** The effect of OTUB1 knockdown on FOXM1 protein expression in Caki-1 and 786-O cells under CHX treatment. **e** The effect of OTUB1 knockdown on FOXM1 protein expression in Caki-1 and 786-O cells under MG132 treatment

OTUB1-mediated deubiquitination of FOXM1 was then investigated in RCC. Knockdown of OTUB1 had no significant effect on FOXM1 mRNA expression (Fig. 3a), while decreased FOXM1 protein expression in Caki-1 and 786-O cells (Fig. 3b). In vivo ubiquitination assay showed that OTUB1 knockdown drastically promoted ubiquitination of FOXM1 (Fig. 3c). We then applied cycloheximide (CHX), a protein synthesis inhibitor in eukaryotic cells, in Caki-1 and 786-O cells transfected with siOTUB1. The result revealed that CHX treatment promoted the decrease of FOXM1 protein, and the decrease rate of FOXM1 was increased in cells transfected with siOTUB1 (Fig. 3d), suggesting that OTUB1 knockdown suppressed the stability of FOXM1. In addition, the stability of FOXM1 was restored in cells transfected with siNC or siOTUB1 under treatment of proteasome inhibitor MG132 (Fig. 3e), showing that FOXM1 protein was decreased by siOTUB1 in a proteasome-dependent manner. Taken together, these results revealed that OTUB1 suppressed ubiquitination of FOXM1 in RCC.

### FOXM1 regulated ECT2-rho signaling

**Fig. 4:**
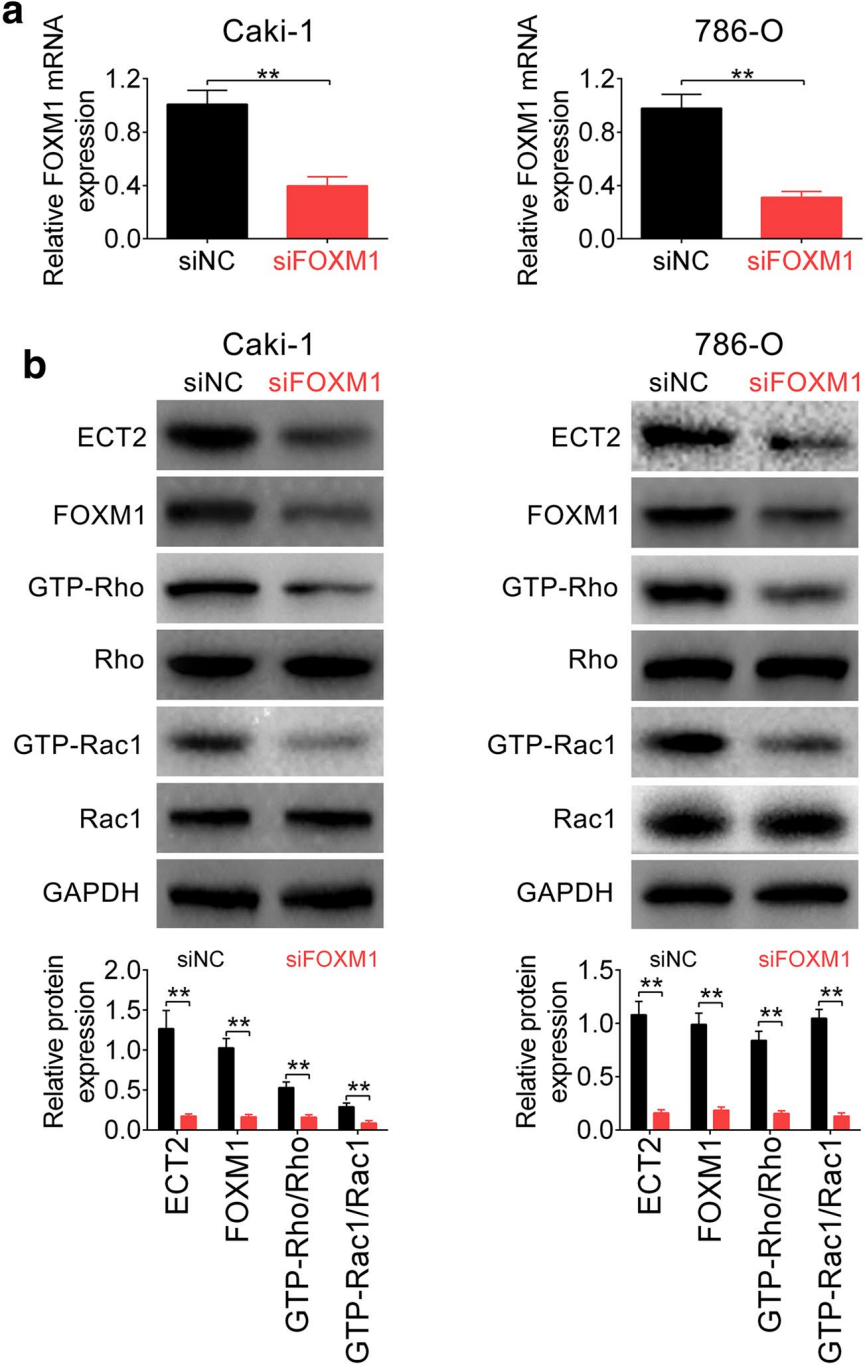
FOXM1 regulated ECT2-Rho Signaling. **a** Knockdown efficiency of siFOXM1 in Caki-1 and 786-O cells as measured by qRT-PCR. ** represents siFOXM1 vs. siNC, *p* < 0.01. **b** The effect of FOXM1 knockdown on proteins expression of ECT2, FOXM1, Rho, GTP-Rho, Rac1 and GTP-Rac1 in Caki-1 and 786-O cells. ** represents siFOXM1 vs. siNC, *p* < 0.01

The downstream target for FOXM1 in RCC was then determined via loss-of function assay. Two siRNAs targeting FOXM1 (siFOXM1 #1 and #2) were designed to knock down expression of FOXM1 and both of them efficiently reduced FOXM1 protein expression (Additional file 1: Figure S1E). SiFOXM1 #1 was named as siFOXM1 and selected for the subsequently functional assays. Knockdown of FOXM1 by siFOXM1 at mRNA level was confirmed in Fig. 4a. Western blot analysis indicated that Caki-1 and 786-O cells transfected with siFOXM1 decreased ECT2 expression compared to cells transfected with siNC (Fig. 4b). Rho signaling, controlled by ECT2 and fundamental for cell migration and invasion, was then investigated. Proteins involved in Rho signaling, Rho and Rac1, were not altered by knockdown of FOXM1 (Fig. 4b). However, the GTP-loaded active Rho and Rac1 were decreased in cells transfected with siFOXM1 (Fig. 4b), suggesting that FOXM1 regulated ECT2-Rho Signaling to participate in RCC migration and invasion. Moreover, siFOXM1 #2 also decreased protein expression of FOXM1, ECT2, GTP-Rho and GTP-Rac1 (Additional file 1: Figure S1E).

### Inhibition ability of OTUB1 knockdown on RCC progression was reversed by FOXM1 over-expression

**Fig. 5:**
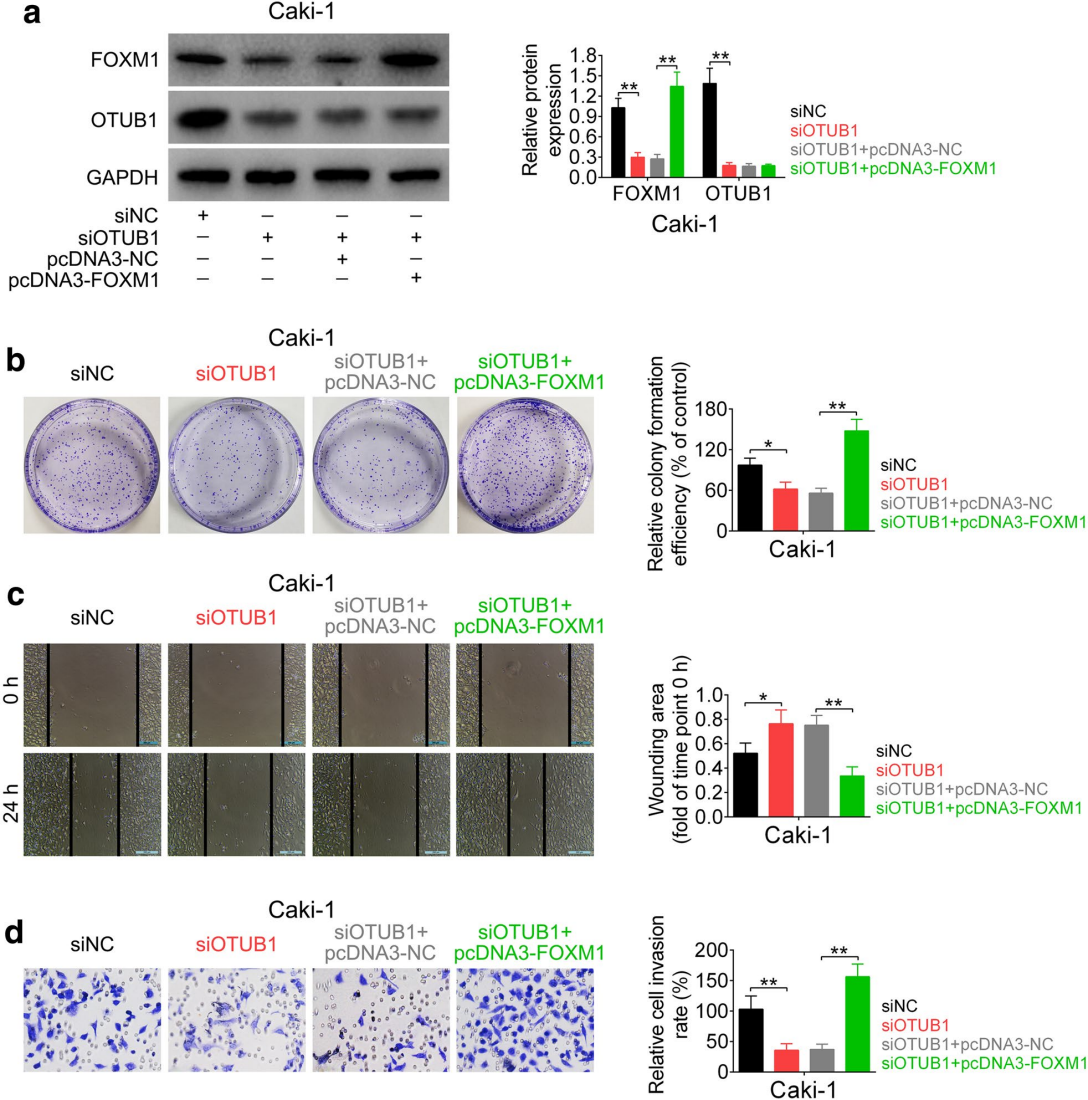
Inhibition ability of OTUB1 knockdown on RCC progression was reversed by FOXM1 over-expression. **a** The effect of siOTUB1 and pcDNA 3.1-FOXM1 on protein expression levels of OTUB1 and FOXM1 in Caki-1 and 786-O cells. ** represents siOTUB1 vs. siNC or pcDNA 3.1-FOXM1 + siOTUB1 vs. siOTUB1 + pcDNA 3.1-NC, *p* < 0.01. **b** The effect of siOTUB1 and pcDNA 3.1-FOXM1 on cell proliferation of Caki-1 and 786-O cells. *, ** represents siOTUB1 vs. siNC or pcDNA 3.1-FOXM1 + siOTUB1 vs. siOTUB1 + pcDNA 3.1-NC, *p* < 0.05, *p* < 0.01. **c** The effect of siOTUB1 and pcDNA 3.1-FOXM1 on cell migration of Caki-1 and 786-O cells. *, ** represents siOTUB1 vs. siNC or pcDNA 3.1-FOXM1 + siOTUB1 vs. siOTUB1 + pcDNA 3.1-NC, *p* < 0.05, *p* < 0.01. **d** The effect of siOTUB1 and pcDNA 3.1-FOXM1 on cell invasion of Caki-1 and 786-O cells. ** represents siOTUB1 vs. siNC or pcDNA 3.1-FOXM1 + siOTUB1 vs. siOTUB1 + pcDNA 3.1-NC, *p* < 0.01

To establish whether FOXM1 is required for OTUB1-mediated RCC progression, Caki-1 cells were co-transfected with siOTUB1 and pcDNA 3.1-FOXM1. FOXM1 was decreased in cells transfected with siOTUB1. Co-transfection of siOTUB1 and pcDNA 3.1-FOXM1 reversed the decrease of FOXM1 (Fig. 5a). Colony formation assay showed that the inhibition ability of siOTUB1 on cell proliferation of RCC was reversed by co-transfection of pcDNA 3.1-FOXM1 (Fig. 5b). Moreover, data from cell migration (Fig. 5c) and invasion (Fig. 5d) analysis indicated that the suppression abilities of siOTUB1 on cell migration and invasion were also suppressed in cells co-transfected with siOTUB1 and pcDNA 3.1-FOXM1. All these results indicated that inhibition ability of OTUB1 knockdown on RCC progression was reversed by FOXM1 over-expression, confirming the role of OTUB1/FOXM1 axis on the regulation of RCC progression.

### OTUB1 knockdown inhibited in vivo RCC tumor growth

**Fig. 6:**
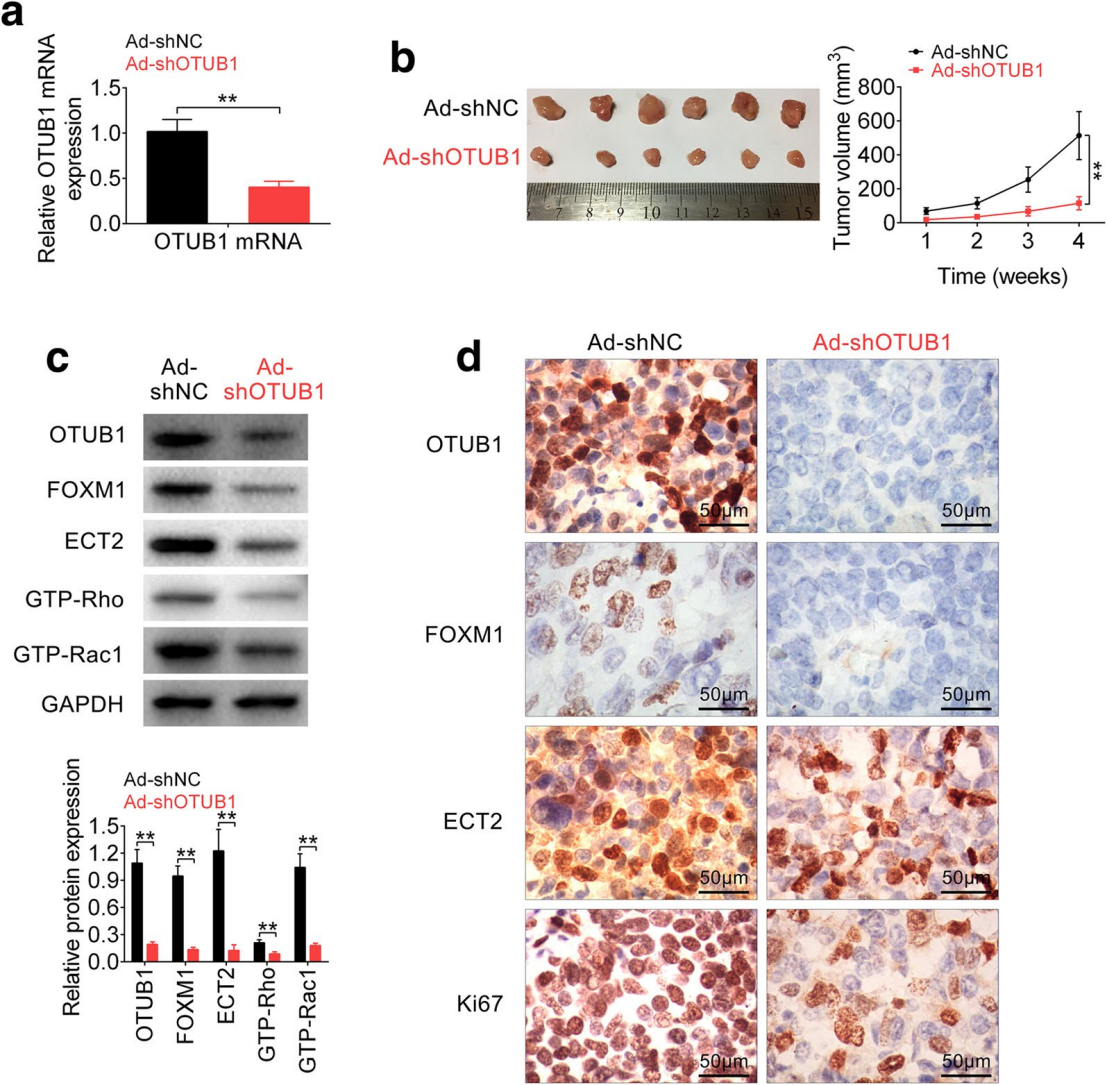
OTUB1 knockdown inhibited in vivo RCC tumor growth. **a** Knockdown efficiency of Ad-shOTUB1 in nude mice as measured by qRT-PCR. ** represents Ad-shOTUB1 vs. Ad-shNC, *p* < 0.01. **b** The effect of Ad-shOTUB1 on RCC tumor growth and volume. ** represents Ad-shOTUB1 vs. Ad-shNC, *p* < 0.01. **c** The effect of Ad-shOTUB1 on proteins expression of OTUB1, FOXM1, ECT2, GTP-Rho and GTP-Rac1 in xenograft tumor mice. ** represents Ad-shOTUB1 vs. Ad-shNC, *p* < 0.01. **d** Immunohistochemistry analysis of OTUB1, FOXM1, ECT2 and Ki67 in tissues of mice intratumorally injected with Ad-shOTUB1 or Ad-shNC

The in vivo xenograft model via inoculation of Ad-shOTUB1 into nude mice was constructed to investigate clinical application of OTUB1 knockdown in RCC. Down-regulation of OTUB1 via Ad-shOTUB1 was confirmed in Fig. 6a. Moreover, the injection of Ad-shOTUB1 inhibited tumor growth (Fig. 6b), as shown by decrease of tumor weight and volume. Furtherly, proteins expression of OTUB1, FOXM1, ECT2, GTP-Rho and GTP-Rac1 were all decreased by Ad-shOTUB1 (Fig. 6c), and immunohistochemistry also indicated the down-regulation of OTUB1, FOXM1, ECT2 and Ki67 in tissues of mice injected with Ad-shOTUB1 (Fig. 6d). However, total protein level of Rho and Rac1 was not affected by Ad-shOTUB1 (Additional file 1: Figure S1F). These results suggested that OTUB1 knockdown inhibited xenograft tumor growth via regulation of FOXM1-mediated ECT-Rho signaling.

## Discussion

OTUB1, as a member of DUBs, regulates ubiquitination and stabilization of tumorigenesis associated proteins such as p53 [28], estrogen receptor α [29], and SMAD2/3 to participate in tumor progression. Moreover, the tumorigenesis associated proteins p53 [30], estrogen receptor α [31] and SMAD2/3 [32] further regulate FOXM1 expression in various tumors, suggesting the critical role of OTUB1/FOXM1 axis in promotion of tumor progression. Considering that FOXM1 is upregulated in many tumor types [33], and FOXM1 participates in RCC progression [34–36], OTUB1/FOXM1 axis may have broad role in tumor progression across multiple tumor types, especially in RCC. Here our study showed, for the first time, that OTUB1 catalyzed deubiquitination and stabilization of FOXM1 to promote RCC progression.

An elevation of OTUB1 expression was firstly found in the present study and shown to be associated with poor prognosis of RCC, suggesting a potential ability of OTUB1 as a prognostic biomarker for RCC. However, due to the small sample size of current clinical analysis (N = 67) between OTUB1 and RCC, the association of OTUB1 expression level with other clinicopathological features of RCC patients may not be precise enough. A larger patient cohort is needed to strengthen the clinical significance of OTUB1 in RCC patients.

Inhibition of DUBs has been shown to affect the ubiquitination and stabilization of DUB-regulated oncoproteins [37], thus leading to tumor growth inhibition [38]. Therefore, DUBs inhibition has been regarded as a novel potential cancer therapeutic strategy. In line with the clinical results of OTUB1 in RCC, in vitro functional assays revealed that knockdown of OTUB1 inhibited cell proliferation, migration and invasion of RCC cells. Moreover, in vivo subcutaneous xenotransplanted tumor model also indicated that knockdown of OTUB1 could suppress in vivo tumorigenic ability of RCC. In conclusion, OTUB1 may not only function as a potential biomarker for RCC diagnosis, but also serve as a potential novel target for RCC therapy.

The underlying mechanism involved in the regulation of RCC progression via OTUB1 was then investigated in the present study. OTUB1 was reported to inhibit the ubiquitination of FOXM1 in ovarian cancer [27] and breast cancer [26]. In line with these studies, our results showed that knockdown of OTUB1 promoted the ubiquitination of FOXM1 in RCC, and the reduction of FOXM1 by protein synthesis inhibitor (CHX) treatment was accelerated via siOTUB1. Moreover, OTUB1 generally restricts the ubiquitination of target proteins in proteasome-dependent manner [39]. Our results also showed that proteasome inhibitor (MG132) treatment increased the stability of FOXM1 in RCC. Furthermore, proteasome-dependent degradation is always associated with proteins with Lys48-linked polyubiquitin chains [40], and OTUB1 prefers target proteins with polyubiquitin chains joined by Lys48 [41], and catalyzes cleavage of the Lys48-linked polyubiquitin chains from FOXM1 [26, 27]. The Lys48-linked polyubiquitin chains deubiquitination of FOXM1 via OTUB1 in RCC needs to be further investigated.

Functional assays indicated that inhibition ability of OTUB1 knockdown on RCC progression was reversed by FOXM1 over-expression, suggesting OTUB1/FOXM1 axis plays a role on the regulation of RCC progression. Although FOXM1 has been shown to participate in RCC progression [25, 34–36], the downstream target is yet to be reported. Here, we found out that ECT2-Rho signaling was involved in the regulation of OTUB1/FOXM1 in RCC. Polo-like kinase 1 (PLK1) phosphorylates FOXM1 to regulate G2/M transition of mitotic cell cycle in RCC [34]. Moreover, PLK1 also phosphorylates ECT2 to regulate G2/M transition of mitotic cell cycle [42]. ECT2 is over-expressed in various tumors and functions as an oncoprotein to promote tumor progression. Knockdown of ECT2 inhibited tumor cell proliferation, migration and invasion [43]. The present study showed that knockdown of FOXM1 decreased ECT2 expression in RCC, thus may inhibit RCC progression. The oncogenic activity of ECT2 works through Rho signaling in breast cancer [44] and hepatocellular carcinoma [45] by transforming inactive GDP-loaded state of Rho to active GTP-loaded state. Our results showed that knockdown of FOXM1 decreased active GTP-loaded state of Rho (GTP-Rho and GTP-Rac1) in RCC, therefore inactivating ECT2-Rho signaling to suppress tumor growth. Moreover, the oncogenic activity of ECT2 also dependents on protein kinase C iota-mediated phosphorylation [46], the effect of OTUB1/FOXM1 axis on regulation of protein kinase C iota needs to be further investigated.

## Conclusion

OTUB1 regulated ubiquitination and stabilization of FOXM1, and OTUB1/FOXM1 axis contributes to RCC tumorigenesis and aggression via ECT2-Rho signaling, suggesting a novel insight into the treatment of RCC.

## Methods

### Patient samples and immunohistochemistry

Surgical cancer or adjacent noncancer specimens from 67 RCC patients were collected at Fujian Medical University Union Hospital. The study was approved by the Ethics Committee of Fujian Medical University Union Hospital, and all the patients signed written informed consent. Paraffined RCC tissues were cut into 4  µm thick sections. The sections were then dewaxed and rehydrated. After washing with PBS (Phosphate Buffered Saline), the sections were blocked with 2% and 0.5% goat serum in PBS, and then incubated overnight with primary rabbit antibodies against OTUB1, FOXM1, ECT2, Ki67 (Abcam, Cambridge, MA, USA). HRP (horseradish peroxidase, Sigma Aldrich, St. Louis, MO, USA)-conjugated goat anti-rabbit IgG secondary antibody was then added to the sections. The slides were counterstained with hematoxylin and examined under light microscope (Olympus, Tokyo, Japan).

### Cell culture

Human RCC cell lines (Caki-1, ACHN, A-498 and 786-O), HK2 (human renal proximal tubular epithelial cell line) and HUVEC (human umbilical vein endothelial cell) were purchased from the Chinese Academy of Sciences (Shanghai, China). All the cell lines were cultured in RPMI-1640 medium (Gibco; Thermo Fisher, Waltham, MA, USA) supplemented with 10% fetal bovine serum at 37 °C constant temperature incubator with 5% CO_2_.

### Cell transfection

siRNAs targeting OTUB1 or FOXM1 as well as the negative control (siNC) were synthesized by GenePharma (Shanghai, China). pcDNA3.1-FOXM1 and the negative control (pcDNA3.1-NC) were obtained from AxyBio co., LTD (Changsha, China). Caki-1 and 786-O cells with 1 × 10^6^ cells/well were seeded into 12-well plate and then transfected with siOTUB1, siFOXM1, siNC, pcDNA3.1-FOXM1 or pcDNA3.1-NC via Lipofectamine^®^ 3000 (Thermo Fisher, Waltham, MA, USA). Two days transfection, the cells were collected for the following experiments.

### Cell proliferation assay

Caki-1 and 786-O cells with 5 × 10^3^ cells/well were seeded in 96-well plates. At 0, 1, 2, 4, 6 days, 20 μL CCK8 solution (Dojindo, Tokyo, Japan) was added into each well and mixed for 3 h. Microplate Autoreader (BioTek, Winooski, VT, USA) was used to measure optical density at 450 nm. For colony formation experiments, Caki-1 and 786-O cells with 200 cells/well were seeded on six-well plate with RPMI 1640 medium. Fourteen days later, the cells were fixed in formalin and stained with crystal violet (0.1%). The visible colonies were counted and photographed under light microscope (Olympus).

### Wound healing and transwell assay

For cell migration analysis, Caki-1 and 786-O cells were seeded in 6-well plates. Wound gap in the cell monolayer was generated by scratching with plastic pipette tip. The cells were washed with PBS to remove debris or the detached cells, and cultured in RPMI-1640 for another 48 h before measuring the wound width. For cell invasion analysis, transfected Caki-1 and 786-O cells were seeded onto the upper wells of chamber (Corning, MA, USA) with the Matrigel-coated membrane (BD Biosciences, Franklin Lakes, NJ, USA) in serum-free RPMI 1640 medium. RPMI 1640 medium with 20% FBS were added to the lower wells. The medium of upper wells and the filters were removed 8 h later. The invasive cells to the bottom of chambers were fixed with 100% methanol and then stained with 0.1% crystal violet 24 h later, imaged and counted under microscope.

### Ubiquitination/deubiquitination and FOXM1 protein turnover assays

Caki-1 and 786-O cells transfected with siOTUB1 or siNC were firstly treated with 10 μM MG132 for 3 h and then collected and lysed. The lysates were immunoprecipitated by anti-FOXM1 (Abcam) and immunoblotted by anti-ubiquitin (Abcam). For measurement of endogenous FOXM1 turnover rate, Caki-1 and 786-O cells transfected with siOTUB1 or siNC were treated with 80 μg/mL cycloheximide (CHX) (Sigma-Aldrich, St. Louis, MO, USA) for inhibition of protein synthesis. At 0, 1, 2, 4 h, cells were harvested and analyzed by western blot.

### Quantitative real-time PCR (qRT-PCR)

Total RNAs from RCC tissues or cell lines were extracted via RNeasy Mini Kit (Qiagen, Manchester, UK). Complementary DNAs were then generated by PrimeScript RT Reagent (Takara, Shiga, Japan). qRT-PCR was analyzed by ViiA 7 (Applied Biosystems, Austin, TX, USA), and the expression fold changes of indicated genes were compared with GAPDH and calculated VIA using 2^−ΔΔCt^ methods. The primer sequences were showed as follows in primer Table 2.

**Table 2:** Primer

ID	Sequence (5′–3′)
GAPDH F	ACCACAGTCCATGCCATCAC
GAPDH R	TCCACCACCCTGTTGCTGTA
OTUB1 F	ACAGAAGATCAAGGACCTCCA
OTUB1 R	CAACTCCTTGCTGTCATCCA
FOXM1 F	ATACGTGGATTGAGGACCACT
FOXM1 R	TCCAATGTCAAGTAGCGGTTG

### Western blot

30 µg proteins from RCC tissues or cells were separated by sodium dodecyl sulfate-polyacrylamide gel electrophoresis, and then transferred to nitrocellulose membrane (Millipore, Bedford, MA). The membranes were blocked by 5% skimmed milk and then incubated overnight with primary antibodies: anti-OTUB1, anti-FOXM1 antibodies (1:1500, Abcam), ECT2 (1:2000, Abcam), GTP-Rho, Rho, GTP-Rac1 and Rac1 (1:2500, Abcam), GAPDH (1:3000, Abcam) at 4 °C. Lastly, the immunoreactivities were detected by enhanced chemiluminescence (KeyGen, Nanjin, China) after incubating with HRP labeled secondary antibody (1:5000; Abcam).

### Mouse xenograft assay

Twelve four-to-five week old female BALB/c nude mice with 18–20 g were purchased from the Animal Center of Wenzhou Medical University (Wenzhou, China), and then separated into two groups. The experimental procedures were conducted in accordance with the guidelines set out by Ethics Committee of the Fifth Medical Center of PLA General Hospital. Ad-shOUTB1, as well as the negative control (Ad-shNC), were constructed by GenePharma (Shanghai, China). 100 μL 1 × 10^9^ transducing units Ad-shNC or Ad-shOTUB1 were injected into the flank regions of nude mice. Tumors were measured with digital calipers every week and the tumor volume was calculated. Four weeks later, mice were anesthetized with 65 mg per kg body weight of sodium pentobarbital, and the xenograft tissues were collected for analysis.

### Statistical analysis

The data were shown as mean ± standard deviation, and the statistics analysis was performed by the SPSS 19.0 (SPSS, Chicago, IL). Student’s t text was used to compare the difference between two groups, one-way ANOVA with Turkey’s test to compare the difference among multiple groups. *P* < 0.05 was considered as statistically significant.

## Supplementary information

**Additional file 1: Figure S1. A** Knockdown efficiency of siOTUB1 #1 and #2 in Caki-1 cells, and the effect of OTUB1 knockdown on protein expression of FOXM1. *, ** represents siOTUB1 vs. siNC, *P* < 0.05, *P* < 0.01. **B** The effect of OTUB1 knockdown on cell viability of Caki-1 cells. * represents siOTUB1 vs. siNC, *P* < 0.05. **C** The effect of OTUB1 knockdown on cell proliferation of Caki-1 cells. ** represents siOTUB1 vs. siNC, *P* < 0.01. **D** The effect of OTUB1 knockdown on cell migration of Caki-1 cells. ** represents siOTUB1 vs. siNC, *P* < 0.01. **E** The effect of FOXM1 knockdown on protein expression levels of ECT2, FOXM1, GTP-Rho and GTP-Rac1 in Caki-1 cells. ** represents siFOXM1 vs. siNC, *P* < 0.01. **F** The effect of Ad-shOTUB1 on protein expression levels of GTP-Rho, Rho, GTP-Rac1 and Rac1 in xenograft tumor mice. ** represents Ad-shOTUB1 vs. Ad-shNC, *P* < 0.01.

## Data Availability

All data generated or analyzed during this study are included in this published article.

## Abbreviations

CCK8: Cell Counting Kit-8
DUBs: Deubiquitinating enzymes
ECT2: Epithelial cell transforming 2
FOXM1: Forkhead box M1
