# Supplementary figures and images for: OTUB1-mediated deubiquitination of FOXM1 up-regulates ECT-2 to promote tumor progression in renal cell carcinoma
Source: Cell Biosci. 2020 Mar 30;10:50. doi: 10.1186/s13578-020-00408-0 (PMC7106863; doi:10.1186/s13578-020-00408-0)

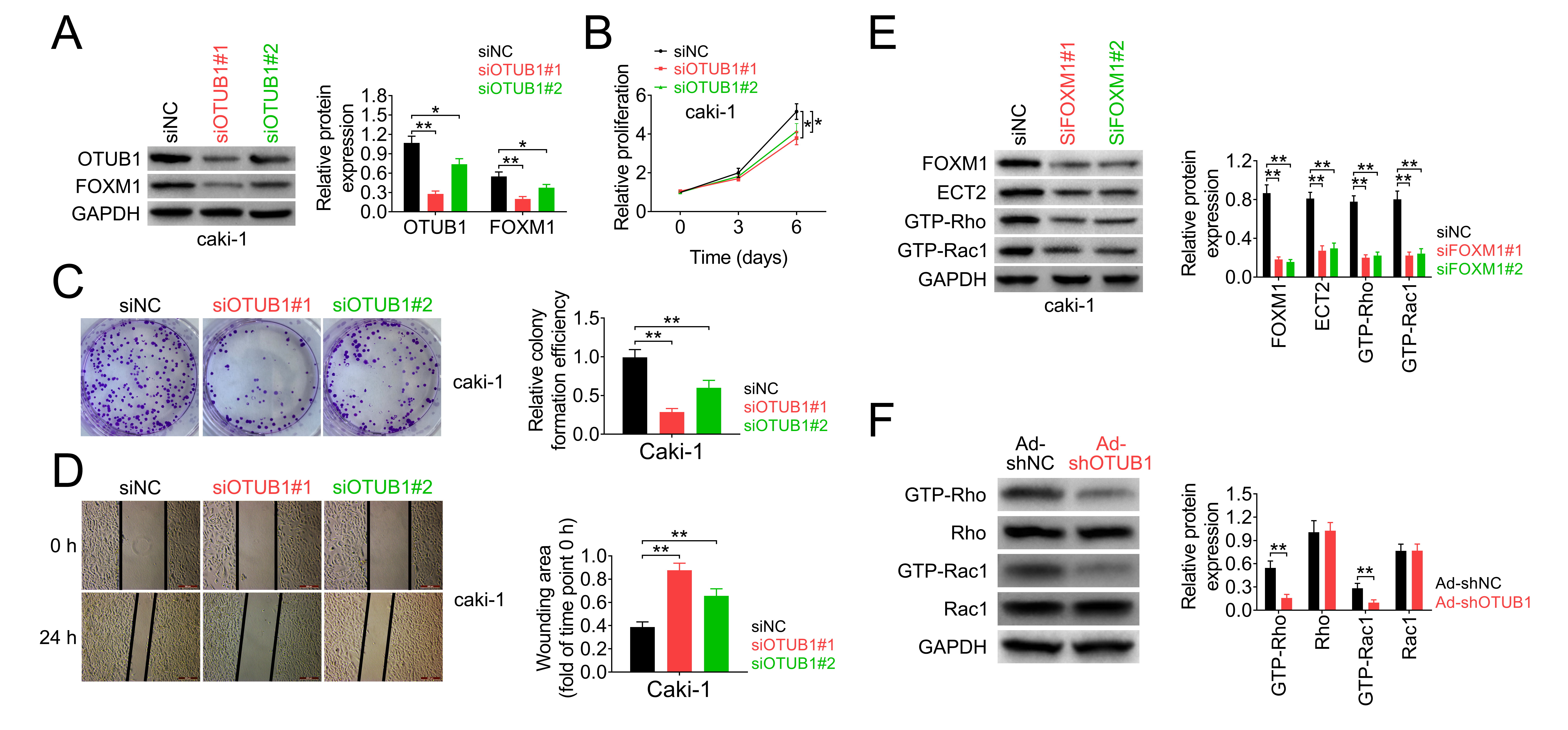

Supplement: Supplementary file 1 — Additional file 1: Figure S1. A Knockdown efficiency of siOTUB1 #1 and #2 in Caki-1 cells, and the effect of OTUB1 knockdown on protein expression of FOXM1. *, ** represents siOTUB1 vs. siNC, P < 0.05, P < 0.01. B The effect of OTUB1 knockdown on cell viability of Caki-1 cells. * represents siOTUB1 vs. siNC, P < 0.05. C The effect of OTUB1 knockdown on cell proliferation of Caki-1 cells. ** represents siOTUB1 vs. siNC, P < 0.01. D The effect of OTUB1 knockdown on cell migration of Caki-1 cells. ** represents siOTUB1 vs. siNC, P < 0.01. E The effect of FOXM1 knockdown on protein expression levels of ECT2, FOXM1, GTP-Rho and GTP-Rac1 in Caki-1 cells. ** represents siFOXM1 vs. siNC, P < 0.01. F The effect of Ad-shOTUB1 on protein expression levels of GTP-Rho, Rho, GTP-Rac1 and Rac1 in xenograft tumor mice. ** represents Ad-shOTUB1 vs. Ad-shNC, P < 0.01. [file 13578_2020_408_MOESM1_ESM.tif]
